# Schnellschnittdiagnostik bei Tumoren des Trigonum submandibulare

**DOI:** 10.1007/s00106-022-01240-3

**Published:** 2022-11-14

**Authors:** S. Riemann, A. Knopf

**Affiliations:** 1 Klinik für Hals‑, Nasen- und Ohrenheilkunde, Universitätsklinikum Freiburg, Medizinische Fakultät, Albert-Ludwigs-Universität Freiburg, Killianstraße 5, 79106 Freiburg, Deutschland; 2 Klinik für Hals‑, Nasen- und Ohrenheilkunde, Klinikum rechts der Isar, Technische Universität München, München, Deutschland

**Keywords:** Lymphknotenexzision, Lymphknotenmetastasen, Speicheldrüsentumoren, Neck-Dissection, Glandula submandibularis, Lymph node excision, Lymph node metastasis, Salivary gland neoplasms, Neck dissection, Submandibular gland

## Abstract

**Hintergrund:**

Die Diagnostik und Therapie von submandibulär gelegenen Läsionen ist eine Herausforderung. Die häufig vorkommenden Karzinome sollten hinreichend sicher operativ entfernt und gleichsam funktionelle Strukturen zuverlässig geschützt werden. Insbesondere der Erhalt neuronaler Strukturen bei gleichzeitiger Tumorkontrolle lässt einzeitige Konzepte zweckdienlich erscheinen, wenngleich diese nicht hinreichend etabliert sind. Ziel der Studie war die Beurteilung der intraoperativen Schnellschnittdiagnostik als mögliche Grundlage eines einzeitigen chirurgischen Therapiekonzepts und somit die Verhinderung funktioneller Alterationen durch Reoperationen.

**Methoden:**

Es wurden 114 konsekutive Patient*innen in die Studie eingeschlossen, bei denen nach der HNO-Spiegeluntersuchung und bildgebender Diagnostik die Artigkeit der Läsion nicht zugeordnet werden konnte. Patient*innen mit bekanntem Karzinom in der Vorgeschichte oder Hinweis auf eine akut entzündliche/karzinogene Primärläsion in der HNO- und/oder bildgebenden Untersuchung wurden ausgeschlossen. Es wurde eine intraoperative Schnellschnittdiagnostik mit der Fragestellung nach Vorliegen eines Karzinoms durchgeführt. Patient*innenbezogene Daten sowie die Zuverlässigkeit des Schnellschnittergebnisses wurden vergleichend erfasst.

**Ergebnisse:**

Bei insgesamt 114 Patient*innen wurde ein intraoperativer Schnellschnitt durchgeführt. Karzinome wurden mit einer Sensitivität von 87 % und einer Spezifität von 100 % diagnostiziert. Die durchgeführte Diagnostik hätte somit dazu geführt, dass in einem einzeitigen Konzept kein Patient unnötig radikal operiert worden wäre. Bei 26 von 30 Patient*innen hätte ein Zweiteingriff vermieden werden können.

**Schlussfolgerung:**

Die intraoperative Schnellschnittdiagnostik stellt potenziell eine wichtige Methode dar, um Karzinome histologisch zu bestätigen und zweizeitige Vorgehen zu vermeiden. Das Fehlen falsch-positiver Befunde hätte ein unerwünscht radikales Vorgehen bei 100 % der Patient*innen verhindert.

## Abstract (en)

**Background:**

Diagnosis and treatment of submandibular lesions is challenging. Carcinomas, which frequently occur, should be surgically removed with sufficient safety margins, while simultaneously protecting functional structures. In particular, the preservation of neuronal structures with simultaneous tumor control suggests that single-stage concepts are appropriate, although they are not yet established. The aim of this study was to evaluate intraoperative frozen section biopsy as a possible basis for a one-stage surgical concept to thus prevent functional alterations by reoperation.

**Methods:**

A total of 114 consecutive patients with neoplastic lesions in whom the type of lesion could not be determined after ENT examination and imaging were included in the study. Patients with a known history of carcinoma or evidence of an acute inflammatory or carcinogenic primary lesion in the ENT examination and/or imaging were excluded. An intraoperative frozen section biopsy was performed to determine the presence of a carcinoma. Patient-related data and the reliability of the frozen section result were recorded comparatively.

**Results:**

Intraoperative frozen section biopsy was performed in a total of 114 patients. Carcinomas were diagnosed with a sensitivity of 87% and a specificity of 100%. There would thus not have been unnecessary radical surgery in a one-stage approach. In 26 of 30 patients with carcinoma, a second operation could have been avoided.

**Conclusion:**

Intraoperative frozen section biopsy is potentially an important method to histologically confirm carcinomas and avoid two-stage procedures. The absence of false-positive findings would have prevented an unnecessary radical procedure in 100% of patients.

## Tumorarten und Therapieansätze

Die Diagnose und Therapie von neoplastischen Läsionen im submandibulären Dreieck ist aufgrund der Vielzahl möglicher Differenzialdiagnosen eine Herausforderung. Sowohl intra- als auch extraglanduläre Läsionen treten hier regelhaft auf. Die engen Nachbarschaftsbeziehungen zum Unterkiefer, Mundboden und der Submandibulardrüse erschweren die Diagnosefindung und Etablierung eines geeigneten therapeutischen Konzepts zudem. Die genaue Diagnose ist jedoch von entscheidender Bedeutung für die Festlegung des geeigneten Umfangs der chirurgischen Behandlung und einer möglicherweise nötigen adjuvanten Therapie. Bei den gutartigen Tumoren der Gl. submandibularis handelt es sich am häufigsten um pleomorphe Adenome [2], während das adenoidzystische Karzinom die häufigste maligne intraglanduläre Neoplasie ist [12, 17]. Bei extraglandulären Läsionen ist die häufigste benigne Diagnose eine Lymphadenitis, während Lymphknotenmetastasen und Lymphome die Mehrzahl der malignen Läsionen ausmachen [11]. Die Inzidenz maligner Tumoren ist deutlich höher als in der Ohrspeicheldrüse, wobei der Anteil in der Literatur mit Werten zwischen 34 und 54 % beziffert wird [8, 11, 14]. Darüber hinaus sind die Empfehlungen für die chirurgische und adjuvante Therapie von Speicheldrüsenneoplasien komplex und z. T. sehr uneinheitlich. Der minimale chirurgische Ansatz, der nur bei niedriggradigen Malignomen angemessen ist, sollte in der vollständigen Entfernung der Drüse und der Lymphknotendissektion im Level I bestehen [21]. Eine adjuvante Strahlentherapie wird vorgeschlagen, wenn eine fortgeschrittene Tumorgröße, eine Einstufung des Differenzierungsgrads als „High-Grade-Form“, regionale Metastasen oder eine perineurale Invasion festgestellt werden [6]. Die adjuvante Therapieeskalation, d. h. eine unnötig intensive Adjuvanz, stellt ein Problem insbesondere bei der Behandlung von Malignomen des submandibulären Dreiecks dar. Dabei hat die R1-Resektion eine besondere Bedeutung. In verschiedenen Studien wurden positive Resektatränder bei etwa 40 % der Patient*innen festgestellt. Diese waren mit einem kürzeren rezidivfreien Überleben [13] und verkürztem Gesamtüberleben verbunden [5, 18]. Nach einer Submandibulektomie, deren finale Histopathologie ein Karzinom ergibt, folgt in aller Regel eine weitere Operation, um Sicherheitsabstände zu vergrößern oder eine Neck-Dissection zu ergänzen. Hierbei führt die fehlende Orientierung allzu häufig zu einem nichtbeurteilbaren R‑Status (Rx) und hiermit zur Intensivierung oder überhaupt erst Notwendigkeit der Adjuvanz [3]. Die Adjuvanz auf Boden des Rx-Status ist unbefriedigend, v. a. im Kontext von Low-Grade-Karzinomen. Darüber hinaus ist die Anatomie des Submandibulardreiecks komplex, und jede Reoperation birgt das Risiko, dass die Resektatränder nicht verbessert werden können, und erhöht gleichzeitig die Gefahr von funktionellen Komplikationen.

Bei klinisch oder sonographisch auffälligen zervikalen Lymphknoten ist die ipsilaterale Neck-Dissection obligatorisch. Das Management des als cN0-Stadium beurteilten Halses wird kontrovers diskutiert [1, 23]. Eine Zusammenfassung von 8 Studien mit insgesamt 147 Patient*innen mit adenoidzystischem Karzinom der Gl. submandibularis, bei denen eine elektive Neck-Dissection der Ebenen I–III durchgeführt wurde, ergab eine okkulte Metastasierungsrate von 29 % [9]. Die Autoren empfahlen daher, dass nach der Diagnose eines malignen Tumors eine Neck-Dissection der Ebenen I, II und III in Kontinuität mit der submandibulären Drüse durchgeführt werden sollte, anstatt nur die Drüse zu entfernen. Es ist daher wichtig, zwischen epithelialen und nichtepithelialen Malignomen im submandibulären Dreieck zu unterscheiden, da z. B. intra- und extraglanduläre Lymphome keinen größeren chirurgischen Ansatz erfordern.

Der Stellenwert eines intraoperativen Schnellschnitts zur Vermeidung von zweizeitigen Operationen ist bislang unklar. Ziel der Studie war die Beurteilung der intraoperativen Schnellschnittdiagnostik als Grundlage eines einzeitigen Therapiekonzepts.

## Methodik

Es wurden 114 konsekutive Patient*innen mit submandibulären Raumforderungen eingeschlossen, bei denen nach stattgehabter HNO-Spiegeluntersuchung und Hals-Sonographie (HR-B-Mode; 14L5-Schallkopf; CDS, farbkodierte Duplexsonographie) kein entzündlicher oder tumoröser Primärbefund oral oder pharyngeal nachgewiesen werden konnte. Patient*innen mit bekannten tumorösen Erkrankungen der Kopf-Hals-Region wurden aus der Studie ausgeschlossen.

Alter, Geschlecht, TNM-Stadium, Resektionsstatus und adjuvante Behandlung wurden retrospektiv für alle Patient*innen erfasst. Es wurden intraoperative Schnellschnitte durchgeführt und in 3 Kategorien unterteilt („Karzinom“, „kein Karzinom“ und „unklar“). Bei allen Patient*innen erfolgte eine endgültige histopathologische Gewebeuntersuchung (HE und Immunhistochemie nach klassischer Einbettung), welche mit den Schnellschnittergebnissen verglichen wurde. Ein positives Ethikvotum der Ethikkommission der Technischen Universität München (Nr. 192/15s) und der Ethik-Kommission der Albert-Ludwigs-Universität Freiburg (Nr. 22-1032) lag vor.

## Ergebnisse

Bei 114 konsekutiven Patient*innen mit submandibulären Tumoren, ohne ersichtlichen entzündlichen oder neoplastischen Primärbefund oral oder pharyngeal, wurde ein intraoperativer Schnellschnitt durchgeführt. Das Durchschnittsalter in dieser Kohorte lag bei 62,2 Jahren, und 61 % der Patient*innen waren Frauen.

**Figure 1:**
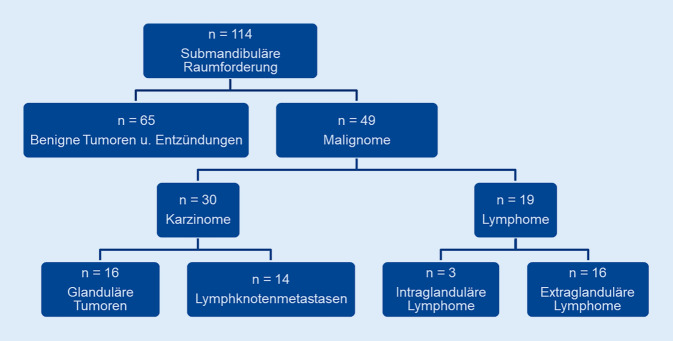

In der endgültigen Histologie wurde bei 65 Patient*innen ein Benignom nachgewiesen, das sich auf unspezifische Lymphadenitiden (*n* = 6) und Sialadenitiden (*n* = 8), mit Immunglobulin G4 (IgG4-)assoziierte Sialadenitiden (*n* = 17) sowie benigne Speicheldrüsentumoren rückbeziehen ließ (Abb. 1 und 2). Demgegenüber wurde bei 49 Patient*innen in der endgültigen Histologie ein Malignom diagnostiziert. In 30 der untersuchten Proben zeigten sich Karzinome, hiervon 16 primäre Speicheldrüsenkarzinome und 14 Metastasen. In 19 Proben ließen sich Lymphome nachweisen, von denen 3 intraglandulär und 16 extraglandulär gelegen waren (Abb. 1).

**Figure 2:**
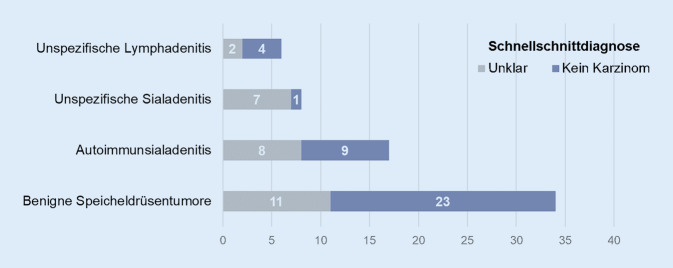

In der intraoperativ durchgeführten Schnellschnittdiagnostik wurde bei 56 Patient*innen (49 %) kein Karzinom festgestellt, während bei 26 Patient*innen (23 %) ein Karzinom mittels Schnellschnitt diagnostiziert wurde. Bei 32 Patient*innen (28 %) konnte im Schnellschnitt keine Diagnose gestellt werden.

Die vorliegende Arbeit sollte die grundlegende Frage beantworten, ob sich die intraoperative Schnellschnittdiagnostik prinzipiell eignet, im Fall eines Karzinoms einzeitige chirurgische Konzepte anzubieten, um den Resektionsstatus (ggf. mit Neck-Dissection) und den Schutz neuronaler Strukturen zu sichern. Hieraus ergeben sich die zentralen Fragen der Sensitivität, also inwieweit Karzinome sicher identifiziert werden, und noch vielmehr der Spezifität, also der sicheren Identifikation nichtkarzinogener Läsionen als Summe der Benignome und Lymphome.

**Figure 3:**
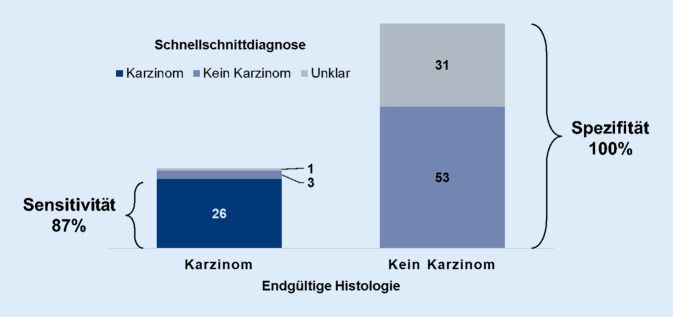

Von 84 nichtkarzinogenen Läsionen wurde im Schnellschnitt keine fälschlicherweise als Karzinom diagnostiziert. Der intraoperative Schnellschnitt erreicht somit eine Spezifität für Karzinome von 100 % (Abb. 3). Da es keine falsch-positiven Schnellschnitte gab, wäre somit kein Patient unnötig radikal operiert worden. Bei allen 26 Tumoren, die im Schnellschnitt als Karzinome klassifiziert wurden, bestätigte die endgültige Histopathologie den Schnellschnitt. Es zeigten sich 13 glanduläre Karzinome und 13 Lymphknotenmetastasen (Abb. 2). Bei 4 Patient*innen konnten Karzinome nicht mittels Schnellschnitt gesichert werden, sondern erst in der endgültigen histologischen Aufarbeitung. Im Schnellschnitt wurden 3 Karzinome als „kein Karzinom“ klassifiziert und eins als „unklar“. Es handelte sich um 2 Karzinome ex pleomorphes Adenom, ein Adenokarzinom und eine Lymphknotenmetastase. Insgesamt wurden 26 von 30 Karzinomen im Schnellschnitt erkannt und daher Karzinome mit einer Sensitivität von 87 % diagnostiziert (Abb. 3).

Gleichsam zeigen sich in der differenzierten Betrachtung die Limitationen der Schnellschnittdiagnostik. Insbesondere die Gruppe der unspezifischen und autoimmunvermittelten Sialadenitiden, aber auch in gewissen Anteilen die gutartigen Speicheldrüsenläsionen erscheinen im Schnellschnitt schwer zuzuordnen und bleiben in diesem häufig unklar (Abb. 3). Mit Blick auf chirurgische Implikationen ergibt sich somit eine klare Gewichtung auf die Spezifität.

## Diskussion

Tumoren des submandibulären Dreiecks stellen eine diagnostische und therapeutische Herausforderung dar. Neben der Vielzahl benigner und maligner Differenzialdiagnosen ist der Anteil an Malignomen hoch. In dieser Studie zeigte sich bei 114 Patient*innen mit submandibulären Tumoren ein Malignomanteil von 43 %. Aufgrund der schwierigen anatomischen Verhältnisse ist ein einzeitiges operatives Konzept für epitheliale Malignome wünschenswert, da jede Zweitoperation die Gefahr der Schädigung wichtiger funktionaler (v. a. neuronaler) Strukturen erhöht. Hier sind v. a. der Ramus marginalis des Gesichtsnervs sowie der sensible und motorische Zungennerv in Gefahr. Tumorresektion und die Neck-Dissection der Level I–III sollten daher bei Karzinomen im Idealfall in einer Operation erfolgen. In dieser Studie wurde gezeigt, dass ein einzeitiges chirurgisches Konzept mittels eines intraoperativen Schnellschnitts möglich ist.

Die Genauigkeit der Schnellschnittpathologie bei Speicheldrüsentumoren wurde in zahlreichen Studien nachgewiesen [2, 15, 16, 19]. In der vorliegenden Studie betrug die Sensitivität 87 % und die Spezifität 100 %, was mit anderen Studien vergleichbar ist [2, 19]. So kann 26 von 30 Patient*innen mit Karzinomen eine zweite Operation erspart werden. Da es keine falsch-positiven Befunde gab, bestand für keine Patient*in in der hier vorgestellten Kohorte das Risiko einer unnötig radikalen Operation.

Für die präoperative Diagnose von Läsionen im submandibulären Dreieck wird häufig die Feinnadelbiopsie verwendet. Die Unzulänglichkeiten dieser Technik wurden in verschiedenen Studien, Übersichtsarbeiten und Metaanalysen aufgezeigt [7, 10, 22]. Die ultraschallgesteuerte Grobnadelstanze kann ein hilfreiches präoperatives Instrument sein, insbesondere, um Operationen bei bestimmten Patient*innengruppen zu vermeiden, z. B. bei Verdacht auf ein Lymphom beim Sjögren-Syndrom [4], obwohl dies für Tumoren der Parotis möglicherweise relevanter ist als für submandibuläre Massen. Insgesamt gibt es nur eine begrenzte Anzahl von Studien, die sich auf Läsionen des submandibulären Dreiecks konzentrieren, und es hat sich gezeigt, dass Studien zu Fein- und Grobnadelbiopsien der Speicheldrüse oft voreingenommen sind [20] und daher die klinische Aussagekraft begrenzt ist. Multimodale Ultraschallalgorithmen können eine ähnliche Sensitivität und Spezifität für die präoperative Diagnose von submandibulären Massen erreichen wie Biopsieverfahren und sind ein wichtiges Instrument, um eine angemessene Patient*innenaufklärung vor der Operation zu ermöglichen [11].

Die komplexe Anatomie des submandibulären Dreiecks birgt außerdem ein Risiko für positive Absetzungsränder, wenn präoperativ kein Malignitätsverdacht besteht. Dieser müsste jedoch allein aufgrund der hohen Malignomrate konsequenterweise angenommen werden. Eine zweite Operation erhöht nicht nur das Risiko für chirurgische Komplikationen, sondern kann auch zu unklaren Absetzungsrändern führen, wenn jene nach der ersten Operation positiv waren. Atula et al. verwendeten Schnellschnitte bei einem Teil ihrer Patient*innen und spekulierten, dass eine häufigere Verwendung einige der zusätzlichen Operationen hätte vermeiden können [2]. Es wurde auch gezeigt, dass positive Schnittränder zu einem verminderten Gesamtüberleben führen [5]. Eine Neck-Dissection im Level I–III kann die Resektatränder verbessern, ohne dass die Morbidität signifikant höher ist als bei einer extrakapsulären Drüsenexzision [14]. Mithilfe der intraoperativen Schnellschnittdiagnostik als Standardvorgehen könnten daher, ohne ein erhöhtes operatives Risiko für die Patient*in, positive Schnittränder vermieden werden.

Zusammenfassend lässt sich sagen, dass die Durchführung einer intraoperativen Schnellschnittuntersuchung dem Chirurgen ermöglicht, Patient*innen mit einem einzeitigen chirurgischen Ansatz zu behandeln. Dies begrenzt das Risiko von Komplikationen und verhindert zweizeitige Operationen bei Patient*innen mit Karzinomen des submandibulären Dreiecks.
